# Reactions on Twitter to updated alcohol guidelines in the UK: a content analysis

**DOI:** 10.1136/bmjopen-2016-015493

**Published:** 2017-02-28

**Authors:** Kaidy Stautz, Giacomo Bignardi, Gareth J Hollands, Theresa M Marteau

**Affiliations:** Behaviour and Health Research Unit, University of Cambridge, Cambridge, UK

**Keywords:** alcohol, alcohol guidelines, health communication, Twitter, social media

## Abstract

**Objectives:**

In January 2016, the 4 UK Chief Medical Officers released a public consultation regarding updated guidelines for low-risk alcohol consumption. This study aimed to assess responses to the updated guidelines using comments made on Twitter.

**Methods:**

Tweets containing the hashtag #alcoholguidelines made during 1 week following the announcement of the updated guidelines were retrieved using the Twitter Archiver tool. The source, sentiment and themes of the tweets were categorised using manual content analysis.

**Results:**

A total of 3061 tweets was retrieved. 6 sources were identified, the most prominent being members of the public. Of 821 tweets expressing sentiment specifically towards the guidelines, 80% expressed a negative sentiment. 11 themes were identified, 3 of which were broadly supportive of the guidelines, 7 broadly unsupportive and 1 neutral. Overall, more tweets were unsupportive (49%) than supportive (44%). While the most common theme overall was sharing information, the most common in tweets from members of the public encouraged alcohol consumption (15%) or expressed disagreement with the guidelines (14%), reflecting reactance, resistance and misunderstanding.

**Conclusions:**

This descriptive analysis revealed a number of themes present in unsupportive comments towards the updated UK alcohol guidelines among a largely proalcohol community. An understanding of these may help to tailor effective communication of alcohol and health-related policies, and could inform a more dynamic approach to health communication via social media.

**Strengths and limitations of this study**

- This is the first study, to the authors' knowledge, to examine responses to an alcohol-related policy announcement using social media content.
- Publicly available comments on social media offer an insight into public responses to policy announcements, as well as being an aspect of the digital environment that may influence the attitudes and beliefs of others.
- The representativeness of Twitter comments is questionable, however, and more work is needed to identify potential sources of biases within social media content.

## Introduction

In January 2016 the four UK Chief Medical Officers issued a public consultation regarding updated guidelines for alcohol consumption, the first time these had been updated since 1995.^1^ Based on expert understanding of the short-term and long-term health risks of alcohol consumption, the new proposed guidelines offer advice for low-risk regular and single occasion drinking. Key points of the updated guidelines include: (1) no level of regular alcohol consumption can be considered as safe in relation to some cancers, as risk increases with any amount consumed; (2) for those choosing to drink alcohol regularly it is safest not to drink more than 14 units of alcohol per week; (3) if drinking within these guidelines, health risks are broadly similar for men and women; and (4) for women who are pregnant or planning a pregnancy it is safest to not drink alcohol at all. In August 2016, in response to the consultation, the final version of the guidelines was released with slightly revised wording. The topic of the current research is the response to revised guidelines as presented in the January announcement of a public consultation, not the response to the amended final version.

**Figure 1:**
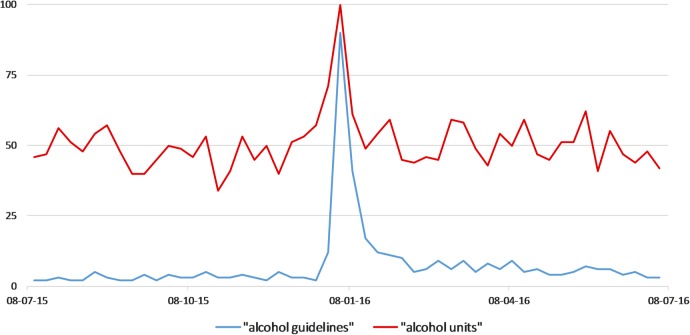
Relative frequency of Google searches for the terms ‘alcohol guidelines’ (blue) and ‘alcohol units’ (red) in the UK from 1 July 2015 to 1 July 2016. The y-axis represents search interest relative to the highest point on the chart. A value of 100 is the peak popularity for the term.

Whether drinkers will heed the updated guidelines is uncertain. In 2007, it was found that fewer than 15% of respondents to the Health Survey for England could correctly define the recommended maximum daily alcohol intake of the time.^2^ More concerning is the observation that many drinkers who can accurately report current drinking guidelines show little intention to drink in accordance with them.^3^ ^4^ Public surveys assessing immediate responses to the announcement of the updated guidelines provide further indication of such reluctance. An online search identified two polls conducted by UK-based regional newspapers on the day the new guidelines were released. The *Belfast Telegraph*^5^ asked readers ‘Will new alcohol guidelines change your habits?’, to which 81% of 215 respondents answered no and 19% answered yes. The *Express & Star*^6^ asked ‘Will you cut your alcohol consumption in light of new guidelines?’, to which the same proportion—81%—of 648 respondents answered no, with 19% answering yes. Despite these negative responses, online search behaviour suggests that the announcement of the revised guidelines successfully generated awareness and interest. Google Trends indicates that the announcement of the revised guidelines led to increased searches for the terms ‘alcohol guidelines’ and ‘alcohol units’. Although the number of searches dropped off substantially in the days following the announcement, there appears to have been a modest increase in searches for ‘alcohol guidelines’ in the 6 months following the announcement, compared with the 6 months prior (figure 1).

A more detailed insight into reactions to the updated guidelines may be gleaned from comments made on the online microblogging community Twitter. Twitter is a rich source of public opinion, with 313 million monthly active users as of June 2016.^7^ Users can post 140 character statements, or tweets, which are presented on that user's profile page and in the content feed of that user's followers, as well as being searchable by other users. Given its large user base and the immediacy of its content, Twitter data can be used to assess responses to news and events, as well as general opinions towards specific topics. Twitter sentiment towards current economic and political issues has been shown to correlate substantially with public opinion gathered from population surveys.^8^ Researchers are beginning to use Twitter content to address health-related questions. For example, public opinion on e-cigarettes, hookah, and cannabis has been characterised using tweets.^9–11^ Regarding alcohol, a content analysis of tweets mentioning alcohol made during 1 month in 2014 found that Twitter chatter about alcohol is overwhelmingly positive, with 79% of tweets being proalcohol and only 7% being antialcohol.^12^

Tweets, like any social media content, are also aspects of the digital environment that might influence attitudes and beliefs.^13^ Social media sites are now a news source for many and for these individuals the first exposure to a story may come infused with the opinions of other users, which may in turn shape opinions and behaviour.^14^ There is evidence linking exposure to alcohol-related content on social media with own alcohol use behaviour. More frequent posting of alcohol-related content by one's friends on social media is associated with one's own alcohol use and clinical symptoms of problematic use,^15^ ^16^ while exposure to any form of alcohol-related media content, including online and social media content, predicts earlier experimentation with alcohol among adolescents.^17^

Twitter content has not yet been used to assess opinions regarding alcohol-related policy, though it has been used to assess opinions and sentiment towards National Health Service reforms in the UK.^18^ The public response to health policy decisions is important and may help to identify issues and improve future health communication. For example, one criticism of the revised guidelines was that they were written with an ‘emphasis on inducing fear through mentions of cancer, and consistent downplaying and even denial of any health benefit’.^19^ Comments made on Twitter may provide evidence pertinent to this criticism. Relatedly, Twitter comments could provide a first insight into whether the revised alcohol guidelines are generating new dialogue about alcohol's negative impact on health, a potential mediating pathway to reducing consumption.^20^

The aim of this study is to describe the source, sentiment and themes of responses to the UK Government's Chief Medical Officers' updated alcohol consumption guidelines using comments made on Twitter.

## Methods

We adhered to recommendations set out by Rivers and Lewis^21^ regarding the collection, analysis and presentation of Twitter data.

### Data source

Public tweets including the hashtag #alcoholguidelines were collected for 1 week from the date the new guidelines were released (8 January 2016) using the Twitter Archiver add-on to Google Sheets.^22^ This tool allows users to download public tweets that include specified hashtags or keywords. Tweets from users who have set their Twitter profiles to be private are not collected.

The first use of the #alcoholguidelines hashtag was by *Good Morning Britain*, a nationally televised morning news and entertainment programme whose Twitter account was followed by around 293 000 users in January 2016. The hashtag was soon picked up by other media outlets and by the UK Department of Health (whose first choice of hashtag, #alcoholupdate, failed to spread throughout the Twitter community), and became the principal tag for discussion about the new guidelines.

Twitter Archiver extracted 3061 original tweets made from 8 to 14 January 2016. These were downloaded on 15 January 2016. The majority of these tweets (2631) were made on the day the new guidelines were released. Retweets, comments reposted by other users with no additional input, were excluded.

### Analytic procedure

#### Spam and irrelevant tweets

We excluded tweets that appeared to be spam, machine-generated (eg, tweets only using the popular hashtag terms of the day), non-sensical or irrelevant to the alcohol guidelines.

#### Source

The source account of each tweet was categorised by viewing each account's screen name, full name and short biography. A list of provisional sources was identified by the first author and refined through discussion between two researchers (KS and GB). To assess the reliability of coding source these two researchers coded a random sample of 100 accounts, which produced a good level of agreement (85%) and a Cohen's κ of 0.62.

#### Sentiment

The sentiment of each tweet was manually coded as either: (1) positive towards the guidelines, (2) negative towards the guidelines, or (3) neutral or communicating no clear sentiment towards the guidelines. Positive or negative sentiment was coded only if the tweet contained sentiment directed specifically towards the guidelines. Tweets that expressed positive or negative sentiment only towards alcohol more generally, for example, were coded as neutral/no sentiment. Coding of a random sample of 100 accounts produced 70% agreement and a Cohen's κ of 0.50.

#### Themes

A list of provisional themes was created by the first author based on an initial viewing of the data, and a preliminary coding scheme was created. Three researchers (KS, GB and GJH) coded a random sample of 150 tweets using this scheme. The number and descriptions of themes and their inclusion criteria were then refined through discussion between these researchers. Two researchers (KS and GB) conducted further iterations of this procedure to develop a detailed coding manual. Once a final list of themes was decided on, 100 tweets were again coded and inter-rater reliability was assessed. The percentage agreement for all themes was high, ranging from 86% to 99%. Cohen's κ was high for five themes, ranging from 0.69 to 0.92. Three themes with weaker κ values (∼0.4) were developed further with more detailed inclusion criteria. Three themes showed poor reliability (<0.3), although these themes had a very low prevalence in the coding sample (0.05–0.14) and therefore high expected chance agreement levels (0.76–0.99), which vastly increases the sampling error of κ.^23^ When the themes and coding manual had been agreed on, two researchers (KS and GB) each coded half of the total tweets. Tweets that expressed multiple themes were coded as such.

## Results

A total of 3061 original tweets from 2291 unique accounts were retrieved. Removal of spam and irrelevant tweets left 2402 tweets from 1856 accounts for analysis. The 437 accounts that only posted irrelevant tweets were not analysed further. A total of 101 tweets (4.2% of the total retained) appeared to be relevant but did not fall into any of the identified themes. These tended to have ambiguous meaning and/or used additional linked images. These tweets were not coded for sentiment.

Of the accounts retained for analysis, most (n=1542, 83.1%) sent only one tweet. The mean tweets per account was 1.29 (SD=0.86). Number of followers of each account ranged from 0 (one account) to 12 277 014. The median number of followers was 487. The collected tweets were retweeted an average of 1.75 (SD=10.50) times and given an average 2.02 (SD=9.20) favourites by other users.

### Source

Six source categories were identified: (1) member of the public (71.1% of tweets, n=1709), (2) health-related organisation or individual (12.4%, n=299), (3) news or media-related organisation or individual (5.8%, n=139), (4) alcohol industry-related organisation or individual (4.0%, n=97), (5) celebrity or public figure (1.3%, n=31), and (6) miscellaneous (5.3%, n=127). Miscellaneous tweets were those that did not fall into any of the other identified categories, examples being businesses and parody accounts.

### Sentiment

The majority of tweets (61.6%, n=1480) were coded as not expressing any specific sentiment towards the guidelines, with 27.4% (n=658) expressing negative sentiment and 6.8% (n=163) expressing positive sentiment.

### Themes

Eleven themes were identified. Table 1 provides a description of each theme, the number of tweets and accounts expressing each theme, and the popularity of these tweets as measured by retweets and favourites by other users. Three themes (1–3 in table 1) were rated as being broadly supportive of the new guidelines, seven (4–10) as broadly unsupportive and one (11) as neutral. Overall there were slightly more tweets that were unsupportive (49.1%) than supportive (43.7%). Tweets within the disagreement theme appeared to be heterogeneous compared with other themes, necessitating further coding into subthemes. Table 2 details these subthemes. The most common were non-specific anger or resistance to the guidelines, and disagreement with the scientific backing of the guidelines.

**Table 1:** Themes identified by content analysis

Theme	Description	Example tweets (paraphrased)	Percentage (number) of tweets expressing theme	Percentage (number) of accounts expressing theme	Mean (SD) retweets	Mean (SD) favourites
*Broadly supportive*						
1. Sharing	Shares recommendations or health information from the guidelines; initiates discussion; provides tips to cut down or stop drinking; links to relevant services or resources	*Read the new alcohol guidelines from Department of Health* *Drink slowly, consume with food, alternate alcohol with water*	30.0% (721)	29.2% (541)	2.96 (12.74)	1.96 (8.11)
2. Agreement	Supports the guidelines; agrees or accepts the need for guidelines; criticises those who are hostile to guidelines	*Guidelines warn about risk of drinking during pregnancy—right to know* *Complaining about #alcoholguidelines? They’re for our own health benefits, so you can make an informed choice*	11.0% (264)	12.9% (239)	1.84 (10.15)	1.53 (5.97)
3. Will heed	Intend to cut down alcohol consumption; no change needed as consumption already within guidelines	*I must limit my intake this weekend. You only get one shot at life!* *14 units a week? PHEW! Should be ok with my bottle of beer on a Saturday night*	2.7% (65)	3.4% (63)	1.00 (5.17)	1.77 (5.00)
*Broadly unsupportive*						
4. You should drink	Encourages others to drink or promotes drinking generally	*If you’re asking is one more drink too much, you’re not drunk enough* *There's “no safe level of drinking” so everybody is getting smashed*	11.9% (285)	14.3% (266)	0.75 (2.16)	1.96 (4.15)
5. Disagreement	General or specific disagreement with the guidelines that does not fall into any other theme	*I don’t trust government advice. How has the research been done? There are so many factors*. *Outrageous to suggest that effects of alcohol on men and women are equal. Absurd!*	11.2% (270)	12.7% (236)	0.82 (3.38)	1.34 (3.07)
6. Will ignore	Will personally ignore the guidelines, consume over the guideline amount or intend to drink alcohol in response	*More noise I’ll ignore, because alcohol is nice* *Tonight I’m going to smash back a bottle of red. Fuck you*	9.5% (228)	11.8% (219)	0.94 (6.89)	2.00 (6.48)
7. Libertarianism	Governments and public bodies should not interfere in private behaviours; advice is untrustworthy; government has ulterior motives for policy decisions	*Sick of being told what to eat and drink* *The nanny state rears its ugly head once again. Why can’t they let people make their own decisions?*	6.2% (149)	7.4% (138)	1.54 (6.30)	1.66 (4.51)
8. Confusion	Confused by the guidelines generally or a specific aspect of them; guidelines will be confusing to others; government advice on alcohol or health is inconsistent	*Red wine is good for you, then it's bad for you, make your mind up!* *They won’t engage the public by referring to “units” rather than commonly understood measures*	4.3% (103)	5.3% (99)	1.80 (12.40)	1.56 (6.42)
9. Fatalism	Death/disease is inevitable no matter what health measures we adopt; alcohol is needed to relieve life's suffering	*You know what? Living puts you in danger of dying* *Enjoy life, ignore the constant health warnings, you are going to die whatever*	3.4% (81)	4.3% (79)	5.00 (30.56)	6.68 (33.53)
10. Won't work	Guidelines will be ineffectual because: people already understand and accept the risk; alcohol use is socially/culturally ingrained; health information does not change behaviour	*People have been drinking alcohol for thousands of years. They won’t stop now* “*I didn’t know alcohol was bad for me! These new guidelines will make me stop immediately!” said nobody*	2.7% (64)	3.3% (62)	0.20 (1.07)	0.92 (2.12)
*Neutral*						
11. Humour	Jokes, sarcasm, wit, but no commentary or opinion on alcohol guidelines	*Can we save up units like people at WeightWatchers save points?* *Drink responsibly—don’t spill your drink!*	9.2% (221)	10.7% (198)	0.74 (1.77)	1.86 (3.59)

**Table 2:** Subcategories of the ‘disagreement’ theme

Subcategory description	Paraphrased examples	Percentage (number) of tweets in disagreement theme expressing subcategory
Anger or resistance towards guidelines but no specific reasons given	*How many more guidelines FFS* *Wish the government and its health minions would keep their advice to themselves*	63.0% (170)
Specific disagreement with the scientific backing of the guidelines	*14 units for BOTH men & women is completely illogical* *Alcohol in moderation actually has a number of health benefits*	18.1% (49)
Guidelines fail to acknowledge pleasure of alcohol use	*Some of my happiest memories were made when I drank over #alcoholguidelines*	7.0% (19)
Guidelines do not go far enough to tackle excessive drinking	*Government should tell the truth that alcohol is poison*	4.8% (13)
Guidelines will negatively impact the economy generally or the alcohol industry specifically	*British pubs have suffered a lot. This is another knife in the pub trade*	2.6% (7)
UK alcohol guidelines differ to other countries	*France has the best guidance on alcohol consumption—none*	1.5% (4)
Miscellaneous		3.3% (9)

**Figure 2:**
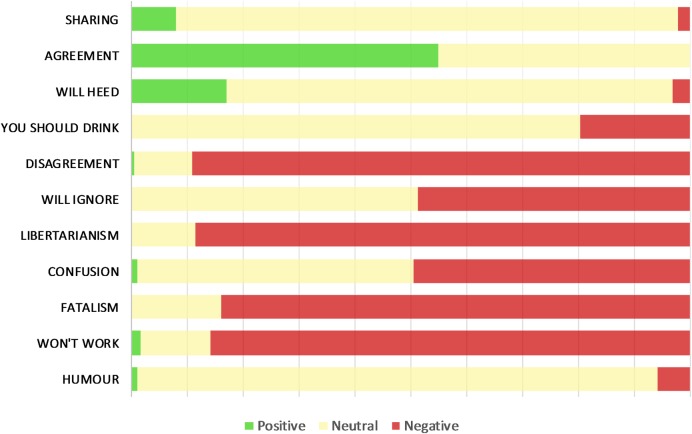
Proportion of positive, neutral and negative sentiment towards the revised guidelines expressed in tweets within each theme.

Levels of sentiment attached to tweets within each theme category varied substantially (figure 2). Many tweets that expressed themes rated as broadly supportive of the revised guidelines did not express positive sentiment. For example, the majority of tweets expressing the sharing theme showed no clear sentiment (89.9%, n=648). Conversely, many of the themes rated as broadly unsupportive did express negative sentiment.

### Comparison of themes expressed by different sources

Table 3 presents a breakdown of sentiment and themes expressed in tweets by each of the six identified sources. A comparison of themes expressed in tweets from the two most prominent sources, members of the public and health-related organisations or individuals, revealed notable differences. The themes most commonly expressed by members of the public in this sample were encouraging others to drink and disagreement. However, sharing information was the third most common theme in this group. Where sentiment towards the guidelines was identified in tweets from members of the public, the majority expressed negative sentiment (34.7% compared with 5.6% expressing positive sentiment). Tweets from health-related accounts were most likely to share information, with the second most common theme being agreement with the guidelines. Tweets from health-related accounts typically expressed no clear sentiment towards the guidelines. Where sentiment was expressed, it was more likely to be positive (15.4% compared with 4.3% negative).

**Table 3:** Proportion (percentage and number) of tweets within each source category expressing sentiment and themes

	Member of the public	Health-related body or individual	News or media-related body or individual	Alcohol industry-related body or individual	Public figure	Miscellaneous
Total tweets	1709	299	139	97	31	127
Sentiment						
Positive	5.6% (95)	15.4% (46)	3.6% (5)	7.2% (7)	3.2% (1)	7.1% (9)
Negative	34.7% (593)	4.3% (13)	10.8% (15)	22.7% (22)	19.4% (6)	7.1% (9)
Neutral/neither	55.2% (943)	78.3% (234)	80.6% (112)	66.0% (64)	61.3% (19)	85.0% (108)
Not coded	4.6% (78)	2.0% (6)	5.0% (7)	4.1% (4)	16.1% (5)	0.8% (1)
Themes						
Sharing	13.8% (235)	85.6% (256)	66.2% (92)	45.4% (44)	6.5% (2)	72.4% (92)
Agreement	11.1% (189)	16.4% (49)	6.5% (9)	9.3% (9)	12.9% (4)	7.1% (9)
Will heed	1.6% (27)	0.3% (1)	0.7% (1)	1.0% (1)	3.2% (1)	0.8% (1)
You should drink	14.5% (247)	1.0% (3)	2.2% (3)	19.6% (19)	16.1% (5)	6.3% (8)
Disagreement	13.9% (237)	2.7% (8)	3.6% (5)	12.4% (12)	9.7% (3)	3.9% (5)
Will ignore	12.2% (208)	1.0% (3)	4.3% (6)	2.1% (2)	19.4% (6)	2.4% (3)
Libertarianism	8.1% (136)	1.0% (3)	2.2% (3)	5.2% (5)	3.2% (1)	0.8% (1)
Confusion	4.9% (84)	2.0% (6)	2.9% (4)	5.2% (5)	3.2% (1)	2.4% (3)
Fatalism	4.6% (78)	0	0	0	3.2% (1)	1.6% (2)
Won't work	3.5% (60)	0	2.2% (3)	1.0% (1)	0	0
Humour	11.4% (195)	1.0% (3)	4.3% (6)	6.2% (6)	12.9% (4)	5.5% (7)
Miscellaneous	4.6% (78)	2.0% (6)	5.0% (7)	4.1% (4)	16.1% (5)	0.8% (1)

### Popularity of tweets by sentiment and theme

Tweets expressing positive sentiment received more retweets (M=1.82, SD=6.45) than negative (M=1.39, SD=11.39) and neutral (M=1.75, SD=9.37) tweets. In contrast, tweets expressing negative sentiment received more favourites (M=2.05, SD=12.21) than those expressing positive (M=1.48, SD=4.60) and neutral (M=1.91, SD=6.69) sentiment.

Point biserial correlations between expression of each theme (coded dichotomously as 0 or 1), and both number of favourites and retweets were calculated, partialling out the number of followers of the tweeting account. Tweets expressing the fatalism theme were significantly positively correlated with both number of favourites (r=0.07, p=0.001) and retweets (r=0.11, p<0.001). There were no other significant correlations.

## Discussion

This study aimed to characterise the response to updated guidelines for alcohol consumption in the UK using publicly available comments made on Twitter. A content analysis of 2402 original and relevant tweets from 1856 unique accounts indicated that tweets came from one of six different sources, with the most common being members of the public and health-related organisations or individuals. Most tweets did not communicate a clear sentiment towards the guidelines. Of the 34% that did, the majority expressed a negative sentiment. Eleven themes were identified, three of which were rated as broadly supportive of the guidelines and seven of which were broadly unsupportive, while one theme, humour, was rated as neutral. The most common theme overall was sharing information. However, most tweets expressing this theme were from health-related sources.

A majority of tweets from members of the public (61%) expressed themes rated as broadly unsupportive of the revised guidelines, with the most commonly expressed theme being encouraging others to drink. The second most common was disagreement, a broad theme that included generalised anger and resistance to the guidelines, disagreements with their scientific backing, and annoyance that the guidelines do not account for the pleasure that alcohol consumption offers. Some of these themes appear to reflect psychological reactance, a commonly observed response to public health warnings regarding alcohol use and other health harming behaviours whereby warnings counterproductively generate cognitions that favour the behaviour being warned against.^24^ ^25^ Such responses are particularly likely among those who engage most heavily in the behaviour.^26^ There is currently limited understanding as to how health communications can be framed to not produce reactance. Encouragingly, however, recent work investigating responses to health warnings on cigarette packaging indicates that such reactance does not hinder behaviour change, and may be a precursor of more deliberative engagement.^27^

Relatedly, many of the unsupportive themes found here offer the opportunity for further engagement with the public and refining of the health messages underpinning the revised guidelines. For example, accounts questioning the guidelines' scientific backing or expressing confusion over aspects of their communication could have feasibly been responded to directly by health professionals. Twitter can be a medium for discussion and public debate, despite tendencies among users to engage in selective exposure and ideological reinforcement.^18^ ^28^ It is notable that while health-related accounts were highly involved in sharing information, there was no evidence of these accounts responding directly to the concerns stated by members of the public. This is a potential utility of using Twitter to communicate health policy that could be explored further.

Regarding the criticism made by the Royal Statistical Society (RSS)^19^ that the revised guidelines may induce fear in the public by focusing on links between alcohol and cancer, none of the themes identified in this analysis reflected fearful responses. However, one subcategory of the disagreement theme did indicate scepticism with the scientific backing of the guidelines, which perhaps supports the RSS's concern that emphasising the negative effects of alcohol while downplaying any positive effects could lead to a loss of public trust in official health guidance. Nonetheless, this subcategory was only evident in 2% of total tweets.

There was notably little sentiment attached to tweets sharing information about the guidelines, or from tweets from health-related accounts in general. While there are advantages to communicating health messages in an ‘affect-free’ manner, these messages were contrasted against many unsupportive tweets that expressed negative sentiment. There is evidence that tweets expressing sentiment are shared more quickly and frequently than neutral tweets.^29^ The use of positive sentiment in health communication on social media could improve its reach. This may be a fruitful area for further research.

### Strengths and limitations

This study is the first, to the best of our knowledge, to examine responses to an alcohol-related policy announcement using social media content. Publicly available tweets offer a large number of potentially useful responses, with few barriers to entry for those wanting to express their views, and with the additional benefit of including immediate affective content.

A key limitation, as with much research using Twitter data, is uncertainty around the representativeness of the users analysed. Our sample comprised a relatively small number of Twitter users, self-selected by the nature of the study, who themselves are only a proportion of internet users. Research into Twitter users from the USA suggests that men and individuals from densely populated areas are over-represented on Twitter, and that the ethnicity of users is not representative of the population.^30^ A further concern is that we were not able to verify whether all tweeters in this sample were expressing their own opinions. It is possible, for instance, that some of the comments were examples of ‘astroturfing’, whereby those with vested interests are involved in propagating fake grass roots opinions in order to sway public debate in their favour.^31^ ^32^ Furthermore, even if comments were the users' own, we are unable to say whether they were responding to the updated guidelines per se, or to reports of the guidelines on other media channels, which may have included provocative comments from alcohol industry representatives. Relatedly, our analysis did not consider the interplay between comments or how themes might have been invoked by the comments of other users in the discussion. Certain themes could have been more likely to be expressed as counterpoints to other themes. A time-based analysis of Twitter dialogue may be a way to address this in future research. Finally, while Twitter comments provide insight into immediate reactions that would not be observable in surveys, they do not indicate how individuals might respond after further deliberation. For example, an immediate negative response to the updated guidelines could have produced motivation to seek further information, which in turn may have changed the initial negative opinion. Nonetheless, immediate affective responses can be important drivers of subsequent decision-making and behaviour.^33^

### Implications for policy

Monitoring of online responses to public health guidance can provide valuable public feedback that may differ with that provided through official consultation. While more work is needed to distinguish sources of bias in comments from non-random samples of Twitter and other social media users, public health bodies responsible for communicating policy announcements could consider monitoring and analysing publicly available comments to learn whether messages are being misunderstood, with a view to clarifying these messages or directly countering misinformation being shared. Social media also provides scope for health professionals to provide dynamic responses to address people's concerns. While some of the themes and subthemes identified reflect emotions or political leanings that might not respond well to further engagement (eg, libertarianism), others may be met quite effectively with further discussion or links to more detailed information.

## Conclusion

Comments made on Twitter offer a potentially valuable source for monitoring responses to health policy announcements. This descriptive analysis of tweets made in response to updated alcohol guidelines in the UK revealed a number of themes present in unsupportive comments towards the revised guidance. An understanding of the reactance, resistance and misunderstanding present in these themes may help to tailor effective communication of alcohol and health-related policies in future, and may inform a more dynamic approach to health communication via social media.
